# Longitudinal Analysis of Diabetes Mellitus Risk: Smoking Status and Smoking Cessation

**DOI:** 10.3390/jcm13133927

**Published:** 2024-07-04

**Authors:** Da-Eun Sung, Seung-Jae Lee, Mi-Yeon Lee, Eun-Jung Rhee, Ki-Chul Sung

**Affiliations:** 1 Department of Internal Medicine, Samsung Medical Center, Sungkyunkwan University School of Medicine, Seoul 06351, Republic of Korea; 2 Division of Cardiology, Department of Internal Medicine, Kangbuk Samsung Hospital, Sungkyunkwan University School of Medicine, Saemunan-ro, Jongno-gu, Seoul 03181, Republic of Korea; 3 Division of Biostatistics, Department of Academic Research, Kangbuk Samsung Hospital, Sungkyunkwan University School of Medicine, Seoul 06351, Republic of Korea; 4 Division Endocrinology and Metabolism, Department of Internal Medicine, Kangbuk Samsung Hospital, Sungkyunkwan University School of Medicine, Seoul 06351, Republic of Korea

**Keywords:** smoking cessation, diabetes mellitus risk, metabolic health, longitudinal analysis

## Abstract

**Background/Objectives**: Smoking cessation is acknowledged for its health benefits. However, it paradoxically increases diabetes mellitus (DM) risk shortly after quitting due to weight gain. This research aimed to investigate how smoking status could affect the development of DM, focusing on how the risk of acquiring diabetes changed over time after quitting smoking, independent of variables such as weight gain. **Methods**: The data of 386,558 participants of the Kangbuk Samsung Health Study, excluding those with pre-existing DM, were examined. Smoking status and its long-term effects on DM risk were assessed using multivariate Cox proportional hazards models. Lifestyle factors, including weight change, physical activity levels, and alcohol intake, were adjusted as time-varying covariates throughout the follow-up period. **Results**: Modified hazard ratios (HRs) indicated no notable disparity in DM risk between individuals who previously smoked and those who had never smoked (HR: 1.04, 95% CI: 0.999–1.08, *p*-value < 0.001). In contrast, current smokers exhibited a significantly increased DM risk (HR: 1.29, 95% CI: 1.24–1.35, *p*-value < 0.001). Within the first six years post-cessation, former smokers initially faced a higher DM risk than never smokers (0–2 years, HR: 1.22, 95% CI: 1.15–1.31, *p*-value < 0.001; 3–5 years, HR: 1.11, 95% CI: 1.04–1.20, *p*-value < 0.001). After 12 years, they realigned with never smokers (12–46 years, HR: 0.92, 95% CI: 0.86–0.98, *p*-value = 0.002). Current smokers consistently showed a higher DM risk (0–9 years, HR: 1.29, 95% CI: 1.14–1.46, *p*-value < 0.001). Adjusting for covariates such as weight change and physical activity did not alter these findings. **Conclusions**: Our results indicated that former smokers initially experienced an elevated risk of DM relative to never smokers. This increased risk aligned with the risk of never smokers after six years, and the risk continued to improve after 12 years compared to never smokers. This contrasted with current smokers, who maintained a heightened risk of DM, even when adjustments were made for weight change, physical activity, and alcohol intake as time-varying covariates.

## 1. Introduction

Smoking cessation is highly recommended as a crucial step to enhance general health and to lower the chances of developing chronic illnesses [1,2]. Despite its extensive benefits, there is a connection between ceasing smoking and the likelihood of developing diabetes mellitus (DM) paradoxically. Although the long-term health advantages of smoking cessation are clear [3,4], studies have indicated a transient rise in the chance of acquiring DM shortly after smoking cessation [5,6,7]. Such a rise is primarily attributed to weight gain [8,9], a common immediate outcome of quitting smoking that is caused by the absence of nicotine’s appetite-suppressing effect.

However, this elevated risk is not permanent. Longitudinal studies have shown that diabetes risk decreases gradually after quitting smoking, eventually reaching the same risk levels as non-smokers [5,6]. Normalization after cessation may take up to ten years, with considerable variation among individuals [10]. This variability underscores the need for a deeper understanding of the mechanisms driving these changes and optimal strategies for mitigating increased diabetes risk during this transitional period.

Despite these insights, the precise time needed for diabetes risk to revert to baseline levels is not clearly established yet. Variations among individuals based on their smoking history and lifestyle changes after quitting are not entirely understood either. Thus, this study aimed to determine the effects of smoking status on the occurrence of DM and the duration required after stopping smoking for the chance of acquiring DM to be equivalent to that of individuals who had never smoked, independent of variables such as weight gain.

## 2. Materials and Methods

### 2.1. Study Population

The study cohort was obtained from the Kangbuk Samsung Health Study (KSHS), a Korean cohort study of participants aged 18 years and above who were undergoing health tests at Kangbuk Samsung Hospital’s Total Healthcare Centers in Seoul and Suwon. This study targeted employees mandated to receive health checks under Korea’s Industrial Safety and Health Law, covering about 80% of the cohort, with the remaining participants being self-enrolled in health screening programs. The KSHS aims to assess risk factors and promote the prevention and early detection of cardiometabolic diseases among other conditions.

**Figure 1:**
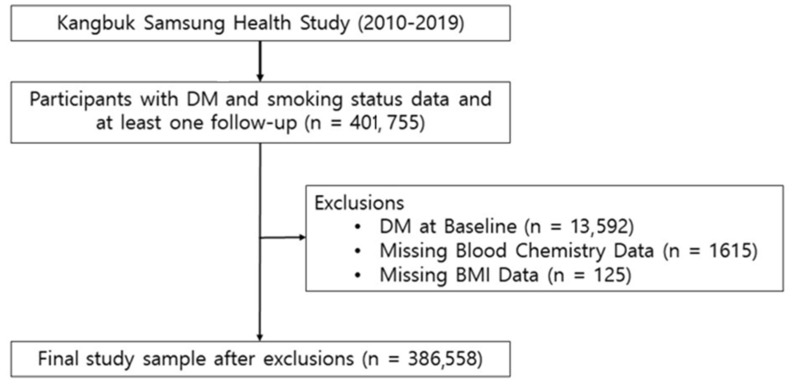
Flow chart of the study participants. DM, diabetes mellitus; BMI, body mass index.

For this analysis, we included participants who completed smoking-related questionnaires from 3 January 2002 to 31 December 2019. We omitted those with pre-existing diabetes mellitus (DM; n = 13,592), those with missing data on blood chemistry (n = 1615), and those with missing data on body mass index (BMI; n = 125), resulting in a final sample of 386,558 subjects (Figure 1). This research received approval from the Institutional Review Board (IRB 2023-07-028) of Kangbuk Samsung Hospital. The need for informed consent was exempted by the IRB because only anonymous and de-identified data were used.

### 2.2. Data Collection

Exams were conducted at the Kangbuk Samsung Health Screening Center following standardized procedures. Serum blood samples of participants were obtained after they fasted for at least 10 h. Analytical procedures were conducted in the Laboratory Medicine Department of Kangbuk Samsung Hospital accredited by the Korean Association of Quality Assurance for Clinical Laboratories and the Korean Society of Laboratory Medicine. All samples were processed and evaluated by the Laboratory Medicine Department at Kangbuk Samsung Hospital [11].

Trained nurses performed anthropometric measurements, such as weight, waist circumference, height, and blood pressure. Waist circumference was measured at the midway between the iliac crest and the lower edge of the rib margin. Blood pressure was taken using an automated oscillometric instrument (53000, Welch Allyn, New York, NY, USA). BMI was determined by dividing weight (in kilograms) by height (in meters) squared.

Hypertension was defined as either a self-reported medical diagnosis, self-reported use of antihypertensive drugs, or the average of three BP measurements with a systolic BP of 140 mmHg or higher or a diastolic BP of 90 mmHg or higher. Diabetes at follow-up was determined by self-reported diabetes history, use of glucose-lowering drugs, HbA1c levels ≥ 6.5% (48 mmol/mol), or fasting glucose levels ≥ 126 mg/dL (7 mmol/L) [12]. Data on medical history and medication usage were collected using a self-administered questionnaire.

Smoking history was obtained at study enrollment using standardized, self-administered questionnaires. Participants were classified into three groups based on their responses—never smokers (individuals who have never smoked), past smokers (individuals who have smoked in the past but had quit smoking at the time of the survey), and current smokers (individuals who were smoking at the time of the survey).

### 2.3. Statistical Analysis

Categorical variables are reported as numerical values and percentages, while continuous variables are shown as mean ± standard deviation (SD) or median [interquartile range (IQR)]. Multivariate Cox proportional hazards regression models were used to obtain hazard ratios (HRs) with 95% confidence intervals (CIs) to investigate the link between long-term smoking and the development of diabetic mellitus (DM). The multivariate model was adjusted for sex, age, hypertension, BMI, regular exercise, education level, high alcohol intake, and fasting glucose. BMI, regular exercise, and high alcohol intake were used as time-varying covariates.

Statistical assessments were conducted using STATA version 16.1 and a *p*-value less than 0.05 was considered to show statistical significance.

## 3. Results

During a median follow-up period of 5.32 years, with an average duration of 6.03 years, DM developed in 19,050 out of 386,558 participants.

Table 1 presents a detailed comparison of baseline characteristics, stratified by DM development status throughout the follow-up period. The mean age of the participants was 36.81 ± 8.12 years. Individuals who developed DM during follow-up were older on average (39.48 ± 8.94 years; *p* < 0.001). The cohort consisted of 54.0% males, with a higher proportion among those who developed DM (76.1% vs. 52.9% in non-DM; *p* < 0.001). The overall mean BMI was 23.18 ± 3.28 kg/m^2^. Participants who developed DM had a significantly higher mean BMI of 25.68 ± 3.53 kg/m^2^ (*p* < 0.001). Higher education was less common among those who developed DM compared to those who did not (56.1% vs. 63.1%). The percentage of those who performed regular exercise was slightly more common in individuals with DM than in those without DM (15.5% vs. 13.9%; *p* < 0.001). This observation may be because individuals with DM are more aware of their risk factors and are thus more likely to engage in regular exercise. However, it may also reflect potential inaccuracies in self-reported exercise data.

The average daily alcohol intake was higher in the DM group than in the non-DM group (8.00 g vs. 5.00 g; *p* < 0.001). Among all participants, 61.3% were never smokers, 17.7% were past smokers, and 20.9% were current smokers.

Utilizing longitudinal data, we assessed the impact of quitting smoking and persisting with smoking on the development of DM (Table 2). Analysis of adjusted hazard ratios (HRs) revealed no significant difference in the risk of developing DM between individuals who had quit smoking and those who had never smoked (HR: 1.04, 95% CI: 0.999–1.08, *p* < 0.001). In contrast, current smokers exhibited a significantly increased DM risk (HR: 1.29, 95% CI: 1.24–1.35, *p* < 0.001).

When the risk of DM development was assessed according to the years of smoking, former smokers initially faced a higher DM risk than never smokers (0–2 years, HR: 1.22, 95% CI: 1.15–1.31, *p* < 0.001; 3–5 years, HR: 1.11, 95% CI: 1.04–1.20, *p* < 0.001), which realigned with the DM risk for never smokers after six years and continued to improve, resulting in an 8% reduction in the DM risk after 12 years compared to never smokers (12–46 years, HR: 0.92, 95% CI: 0.86–0.98, *p* = 0.002) (Table 2). Current smokers consistently showed a higher DM risk (0–9 years, HR: 1.29, 95% CI: 1.14–1.46, *p* < 0.001).

Our analysis included a thorough comparison between Model 3 and Model 4 to assess the effects of time-varying covariates on the risk of DM associated with smoking status. Model 3 was adjusted for sex, age, hypertension (HTN), diabetes mellitus (DM), LDL-C, and alcohol consumption. Model 4 was also adjusted for these factors except that BMI, regular exercise, and high alcohol consumption were also treated as time-varying covariates. In both models, the HR for former smokers remained consistent, showing no substantial variation in DM risk compared to never smokers (adjusted HR: 1.04, 95% CI: 0.999–1.08, *p* < 0.001). Consistent across both models, current smokers exhibited a significantly higher DM risk (adjusted HR: 1.29, 95% CI: 1.24–1.35, *p* < 0.001), demonstrating a strong connection between active smoking and increased DM risk.

Table 3 shows changes in BMI, weight, and waist circumference based on smoking status.

Never smokers exhibited slight increases over time (BMI: 0.18, weight: 0.47, waist: 0.40). Former smokers experienced more pronounced changes in these metrics shortly after cessation, particularly within the first 0–2 years (BMI: 0.22, weight: 0.65, waist: 0.49). These values tended to decrease over time, suggesting a gradual improvement in health metrics as the adverse effects of smoking wane. Specifically, long-term quitters (12–46 years since cessation) demonstrated minimal increases (BMI: 0.06, weight: 0.13, waist: 0.21). In contrast, current smokers, especially those with a smoking duration of 0–9 years, experienced significant rises in these parameters (BMI: 0.35, weight: 1.05, waist: 0.79). Overall, the data indicate that both quitting and continuing smoking influence BMI, weight, and waist measurements, with variations depending on smoking duration and time since cessation.

Baseline characteristics such as education levels, exercise frequency, and alcohol intake showed significant disparities, generally favoring the non-DM group. (Supplementary Table S1).

## 4. Discussion

This study elucidated temporal dynamics of DM risk associated with smoking cessation. Our findings reveal that former smokers exhibited an initial increase in DM risk compared to never smokers. However, this risk normalized after six years and showed further improvement beyond 12 years. This contrasted with current smokers, who maintained a heightened risk of DM, even when adjustments were made for weight change, physical activity, and alcohol intake as time-varying covariates. These findings underscore the persistent adverse effects of active smoking on metabolic health.

Smoking is known to increase resting energy expenditure [13] and elevate plasma leptin levels [14], contributing to reduced body weight. Despite these physiological changes resulting in lower body weights, smokers in our study demonstrated a significantly elevated risk for developing DM. Contrary to expectations that cessation of smoking would mitigate DM risk, we observed a transient elevation in DM risk shortly after cessation. While previous studies often attribute this elevation to weight gain [15], our analysis, adjusted for weight change and other covariates, suggests that intrinsic factors related to smoking itself predominantly drive the temporary increase in DM risk [16,17]. This phenomenon may stem from the extended influence of nicotine on metabolic functions, which can continue to impair glucose regulation and insulin sensitivity for years post-cessation [17]. Moreover, cessation may trigger significant alterations in the inflammatory profile [18,19,20], potentially exacerbating insulin resistance and increasing DM risk during the adjustment period.

Although various studies have investigated lesser-known factors contributing to the onset of diabetes [21,22,23], the relationship between smoking cessation and diabetes has not been thoroughly explored. Additional research is required to explore how changes in lipid profiles, gut microbiota, and hormonal levels associated with stress and weight regulation—such as cortisol, leptin, and ghrelin—affect DM risk after smoking cessation [24,25,26]. Understanding these relationships will enhance our comprehension of the complex interplay between smoking cessation and metabolic health.

Despite the initial increase in DM risk, the alignment of former smokers’ risk with that of never smokers over time supports the substantial long-term health benefits of quitting smoking. This evidence highlights the importance of persistence in cessation efforts and provides a timeline for risk normalization, serving as a motivational tool. Moreover, the variability in DM risk normalization post-cessation [27] underscores the necessity for personalized cessation strategies that consider individual smoking duration, nicotine dependence, and metabolic profiles to effectively manage and reduce elevated DM risks. Our findings challenge the traditional understanding of post-cessation DM risk and underscore the necessity of incorporating these dynamics into patient counseling and DM prevention programs. This study not only enhances our understanding of metabolic outcomes of quitting smoking, but also provides a critical timeline for risk normalization that is essential for optimizing cessation interventions.

### 4.1. Study Strengths and Limitations

The extensive cohort of 386,558 participants enhanced the statistical power and generalizability. Its focus on the temporal dynamics between smoking cessation and DM risk normalization offers valuable insights into the timeframe required for risk alignment with never smokers. Adjusting for multiple confounders, including time-varying covariates such as BMI, allows for a nuanced understanding of direct impacts of smoking cessation. However, several limitations should be noted. Firstly, the reliance on self-reported smoking status introduces potential misclassification bias. Self-reported data are susceptible to recall errors and social desirability biases, which can lead to the incorrect categorization of participants. Secondly, the observational nature of our study and the lack of regular follow-up visits to confirm smoking status over time may limit the accuracy of our findings. Without periodic verification, changes in smoking behavior during the study period might not be fully captured, potentially leading to some misclassification of participants. Additionally, despite defining hypertension as the average of three BP measurements, we could not entirely exclude the possibility of white-coat hypertension. The observation that regular exercise was slightly more common in individuals with DM than in those without DM may reflect an increased awareness of risk factors among individuals with DM or potential inaccuracies in self-reported exercise data.

In summary, addressing these limitations in future research could enhance the accuracy and reliability of the findings. Future studies could mitigate the issue of self-reporting inaccuracies by employing biochemical validation methods, such as measuring cotinine levels, to objectively verify smoking status. Longitudinal studies with regular follow-ups are necessary to accurately classify smoking status and to better understand how changes in smoking behavior over time impact DM risk. Utilizing objective measures of smoking status and ensuring regular follow-up assessments will be crucial for advancing our understanding of the metabolic consequences of smoking cessation and developing effective DM prevention strategies.

### 4.2. Conclusions

Our study elucidated the complex relationship between smoking cessation and DM risk. Former smokers initially experienced an elevated risk of DM relative to never smokers. However, this increased risk matched the risk of never smokers after six years and improved, reducing the DM risk after 12 years. The risk of DM among current smokers remained high even after controlling for weight change, physical activity, and alcohol consumption as time-varying variables. This persistent risk implies that variables other than weight gain can substantially affect metabolic risk.

## Figures and Tables

**Table 1:** Baseline characteristics stratified by DM development status.

	Total	Non-DM	DM	*p*-Value
Number	386,558 (100.0%)	367,508 (95.1%)	19,050 (4.9%)	
Age, years	36.81 ± 8.12	36.67 ± 8.05	39.48 ± 8.94	<0.001
Male, n (%)	208,733 (54.0%)	194,243 (52.9%)	14,490 (76.1%)	<0.001
BMI, kg/m^2^	23.18 ± 3.28	23.05 ± 3.21	25.68 ± 3.53	<0.001
Higher education, n (%)	242,607 (62.8%)	231,921 (63.1%)	10,686 (56.1%)	<0.001
Regular exercise, n (%)	54,109 (14.0%)	51,163 (13.9%)	2946 (15.5%)	<0.001
Alcohol intake, g/day	5.00 (1.00–14.00)	5.00 (1.00–14.00)	8.00 (2.00–20.00)	<0.001
High alcohol intake, n (%)	42,901 (11.1%)	39,965 (10.9%)	2936 (15.4%)	<0.001
Fasting glucose, mg/dL	92.00 (87.00–98.00)	92.00 (87.00–97.00)	102.00 (94.00–110.00)	<0.001
Total cholesterol, mg/dL	190.74 ± 33.28	190.11 ± 33.03	202.87 ± 35.56	<0.001
LDL-C, mg/dL	114.61 ± 30.82	114.12 ± 30.67	124.12 ± 32.04	<0.001
HDL-C, mg/dL	57.86 ± 14.69	58.24 ± 14.72	50.51 ± 11.91	<0.001
Triglycerides, mg/dL	91.00 (64.00–134.00)	89.00 (64.00–131.00)	139.00 (95.00–201.00)	<0.001
HbA1C	5.45 ± 0.29	5.44 ± 0.28	5.71 ± 0.36	<0.001
SBP, mmHg	110.42 ± 13.28	110.03 ± 13.11	118.06 ± 14.17	<0.001
DBP, mmHg	71.00 ± 9.79	70.71 ± 9.68	76.66 ± 10.22	<0.001
HTN	39,151 (10.1%)	34,484 (9.4%)	4667 (24.5%)	<0.001
Smoking status				<0.001
Never smoker	237,151 (61.3%)	229,993 (62.6%)	7158 (37.6%)	
Former smoker	68,443 (17.7%)	63,324 (17.2%)	5119 (26.9%)	
Current smoker	80,964 (20.9%)	74,191 (20.2%)	6773 (35.6%)	

DM = diabetes mellitus; BMI = body mass index; LDL-C = low-density lipoprotein cholesterol; HDL-C = high-density lipoprotein cholesterol; SBP = systolic blood pressure; DBP = diastolic blood pressure; HTN = hypertension. Higher education: college graduate or higher; regular exercise: ≥ 3 time per week; high alcohol intake: >30 g/day (male) and >20 g/day (female).

**Table 2:** Risk of DM development by stratifying long-term smoking status into six groups.

	Number	Event *	HR (Model 1)	HR (Model 2)	HR (Model 3)	HR (Model 4)
Smoking status						
Never smoker	237,151	7158	1 (reference)	1 (reference)	1 (reference)	1 (reference)
Former smoker	68,443	5119	1.1 (1.06–1.15)	1.07 (1.03–1.12)	1.04 (0.999–1.09)	1.04 (0.999–1.08)
Current smoker	80,964	6773	1.39 (1.34–1.45)	1.29 (1.24–1.34)	1.29 (1.24–1.35)	1.29 (1.24–1.35)
Smoking status						
Never smoker	237,151	7158	1 (reference)	1 (reference)	1 (reference)	1 (reference)
Former smoker (0–2 years)	17,890	1206	1.3 (1.22–1.39)	1.21 (1.13–1.3)	1.22 (1.15–1.31)	1.21 (1.13–1.29)
Former smoker (3–5 years)	13,729	948	1.22 (1.14–1.31)	1.15 (1.07–1.23)	1.11 (1.04–1.2)	1.11 (1.03–1.19)
Former smoker (6–8 years)	9225	673	1.16 (1.07–1.26)	1.09 (1.001–1.18)	1.07 (0.98–1.16)	1.06 (0.98–1.15)
Former smoker (9–11 years)	11,019	888	1.08 (1–1.16)	1.05 (0.98–1.13)	0.98 (0.91–1.05)	0.98 (0.91–1.06)
Former smoker (12–46 years)	16,580	1404	0.92 (0.86–0.98)	0.95 (0.9–1.02)	0.92 (0.86–0.98)	0.92 (0.86–0.98)
Current smoker (0–9 years)	6583	284	1.34 (1.19–1.52)	1.22 (1.08–1.38)	1.29 (1.14–1.46)	1.28 (1.13–1.44)
Current smoker (10–14 years)	16,889	817	1.34 (1.24–1.45)	1.19 (1.1–1.29)	1.2 (1.11–1.29)	1.17 (1.08–1.26)
Current smoker (15–19 years)	20,017	1453	1.41 (1.32–1.5)	1.25 (1.18–1.33)	1.24 (1.16–1.32)	1.22 (1.14–1.3)
Current smoker (20–24 years)	20,811	2098	1.46 (1.38–1.54)	1.33 (1.26–1.41)	1.37 (1.29–1.45)	1.37 (1.3–1.45)
Current smoker (25–50 years)	16,664	2121	1.37 (1.3–1.45)	1.34 (1.27–1.42)	1.32 (1.25–1.4)	1.33 (1.26–1.41)

HR, hazard ratio. Model 1 adjusted for age and sex. Model 2 adjusted for age, sex, HTN, BMI, regular exercise, and education level. Model 3 adjusted for age, sex, HTN, BMI, regular exercise, education level, high alcohol intake, and fasting glucose. Model 4 adjusted for age, sex, HTN, education level, fasting glucose, BMI, regular exercise, and high alcohol intake as time-varying covariates. * Event: number of participants who developed incident DM during the study period.

**Table 3:** The slope (beta) for the observations within each study subject.

Smoking Status	Number	Baseline			Change (Slope)					
		BMI, kg/m^2^	Weight, kg	Waist, cm	BMI		Weight		Waist	
					Number	beta	Number	beta	Number	beta
Never smoker	237,151 (61.3%)	22.44 ± 3.19	60.89 ± 11.63	77.51 ± 9.27	237,136	0.18 ± 0.82	237,136	0.47 ± 2.23	229,400	0.40 ± 2.96
Former smoker (0–2 years)	17,890 (4.6%)	24.39 ± 3.12	73.24 ± 11.33	85.11 ± 8.69	17,890	0.22 ± 0.79	17,890	0.65 ± 2.39	17,890	0.49 ± 2.59
Former smoker (3–5 years)	13,729 (3.6%)	24.28 ± 3.07	72.35 ± 11.32	84.53 ± 8.61	13,729	0.17 ± 0.73	13,729	0.51 ± 2.17	13,729	0.43 ± 2.45
Former smoker (6–8 years)	9225 (2.4%)	24.13 ± 3.04	71.60 ± 11.16	84.13 ± 8.54	9225	0.14 ± 0.64	9225	0.40 ± 1.90	9225	0.33 ± 2.19
Former smoker (9–11 years)	11,019 (2.9%)	24.15 ± 2.89	71.40 ± 10.63	84.19 ± 8.21	11,018	0.10 ± 0.55	11,018	0.27 ± 1.66	11,018	0.29 ± 1.96
Former smoker (12–46 years)	16,580 (4.3%)	24.10 ± 2.83	70.64 ± 10.34	84.25 ± 8.00	16,580	0.06 ± 0.56	16,580	0.13 ± 1.65	16,580	0.21 ± 2.04
Current smoker (0–9 years)	6583 (1.7%)	23.98 ± 3.38	71.35 ± 12.84	83.24 ± 9.47	6583	0.35 ± 0.99	6583	1.05 ± 2.95	6583	0.79 ± 3.07
Current smoker (10–14 years)	16,889 (4.4%)	24.32 ± 3.29	73.51 ± 11.90	84.68 ± 9.00	16,889	0.29 ± 0.77	16,889	0.89 ± 2.36	16,888	0.72 ± 2.57
Current smoker (15–19 years)	20,017 (5.2%)	24.54 ± 3.22	74.23 ± 11.45	85.58 ± 8.67	20,017	0.18 ± 0.64	20,017	0.53 ± 1.96	20,015	0.46 ± 2.21
Current smoker (20–24 years)	20,811 (5.4%)	24.56 ± 3.05	73.64 ± 10.72	85.48 ± 8.25	20,811	0.11 ± 0.54	20,811	0.32 ± 1.63	20,810	0.35 ± 1.87
Current smoker (25–50 years)	16,664 (4.3%)	24.47 ± 2.85	72.17 ± 9.89	85.58 ± 7.76	16,664	0.08 ± 0.54	16,664	0.18 ± 1.59	16,664	0.36 ± 2.07

BMI = body mass index.

## Data Availability

The data that support the findings of this study are available from the corresponding author, Ki-Chul Sung, upon reasonable request.

## Supplementary Materials

The following supporting information can be downloaded at: https://www.mdpi.com/article/10.3390/jcm13133927/s1, Table S1: Comparison of the baseline characteristics of males and females in the total population.
