# Supplementary material for: Longitudinal Analysis of Diabetes Mellitus Risk: Smoking Status and Smoking Cessation
Source: J Clin Med. 2024 Jul 4;13(13):3927. doi: 10.3390/jcm13133927 (PMC11242592; doi:10.3390/jcm13133927)
Supplement: Supplementary file 1 [file jcm-13-03927-s001.zip › jcm-3042714-supplementary.pdf]

Supplementary Data

Title: Longitudinal Analysis of Diabetes Mellitus Risk: Smoking Status and Smoking Cessation

Da-Eun Sung [a], Seung-Jae Lee [b], Mi-Yeon Lee [c], Eun-Jung Rhee [d], Ki-Chul Sung [b,*]

Corresponding author: kcmd.sung@samsung.com (K.- C. Sung)

Contents

Supplementary Table S1

Supplementary Table.

Supplementary Table S1. Comparison of the baseline characteristics of male and female the total population

	**Female**	**Male**	**p-value**
**Number**	177,825 (46.0%)	208,733 (54.0%)	
**Age, years**	36.59 ± 8.36	37.00 ± 7.90	<0.001
**BMI, kg/m^2**	21.66 ± 2.98	24.47 ± 2.95	<0.001
**Higher education, n (%)**	101,684 (57.2%)	140,923 (67.5%)	<0.001
**Regular exercise, n (%)**	22,293 (12.5%)	31,816 (15.2%)	<0.001
**Alcohol intake, g/day**	2.00 (0.00 - 5.00)	11.00 (4.00 - 23.00)	<0.001
**High alcohol intake, n (%)**	7,934 (4.5%)	34,967 (16.8%)	<0.001
**Fasting glucose, mg/dL**	90.00 (85.00 - 95.00)	94.00 (89.00 - 99.00)	<0.001
**Total cholesterol, mg/dL**	184.80 ± 32.09	195.80 ± 33.43	<0.001
**LDL-C, mg/dL**	106.27 ± 28.77	121.71 ± 30.72	<0.001
**HDL-C, mg/dL**	64.23 ± 14.81	52.44 ± 12.19	<0.001
**Triglycerides, mg/dL**	72.00 (55.00 - 97.00)	114.00 (81.00 - 164.00)	<0.001
**HbA1C**	5.46 ± 0.28	5.44 ± 0.29	<0.001
**SBP, mmHg**	104.33 ± 12.06	115.62 ± 12.00	<0.001
**DBP, mmHg**	66.63 ± 8.68	74.73 ± 9.12	<0.001
**HTN**	8,733 (4.9%)	30,418 (14.6%)	<0.001
**Smoking status**			<0.001
**Never smoker**	166,537 (93.7%)	70,614 (33.8%)	
**Former smoker**	6,593 (3.7%)	61,850 (29.6%)	
**Current smoker**	4,695 (2.6%)	76,269 (36.5%)	

BMI = body mass index; LDL-C = low-density lipoprotein-cholesterol; HDL-C = high-density lipoprotein-cholesterol; SBP = systolic blood pressure; DBP = diastolic blood pressure; hsCRP = high sensitivity C-reactive protein; HOMA-IR = homeostasis model assessment of insulin resistance; HTN = hypertension; DM = diabetes mellitus.

Higher education : college graduate or higher, Regular exercise : ≥ 3 time per week, High alcohol intake : >30g/day(male) & >20g/day (female)
